# Use of Ca-alginate immobilized *Pseudomonas aeruginosa* for repeated batch and continuous degradation of Endosulfan

**DOI:** 10.1007/s13205-016-0438-2

**Published:** 2016-06-06

**Authors:** Vijayalakshmi Pradeep, Usha Malavalli Subbaiah

**Affiliations:** Department of Life Sciences, SGS, Jain University, JC Road, Bangalore, 560011 India

**Keywords:** *Pseudomonas aeruginosa*, Ca-alginate immobilization, Repeated batch degradation, Continuous degradation, Plasmid curing, PCR analysis, Intracellular degradative enzymes

## Abstract

The current investigation is taken up with the aim of studying repeated batch and continuous degradation of Endosulfan, using Ca-alginate immobilized cells of *Pseudomonas aeruginosa* isolated from an agricultural soil. The work involves the study of genes and enzymes involved in the degradation of the pesticide and was carried out with an objective of reducing the toxicity of Endosulfan by degrading it to less toxic metabolites. The long-term stability of Endosulfan degradation was studied during its repeated batch degradation, carried out over a period of 35 days. Immobilized cells of *Ps. aeruginosa* were able to show 60 % degradation of Endosulfan at the end of the 35th cycle with a cell leakage of 642 × 10^4^ Cfu/mL. During continuous treatment, with 2 % concentration of Endosulfan, 100 % degradation was recorded up to 100 mL/h flow rate and with 10 % concentration of the Endosulfan, and 100 and 85 % degradation was recorded at 20 mL/h flow rate and 100 mL/h flow rate, respectively. After degradation of Endosulfan, products were extracted from a large amount of spent medium using two volumes of ethyl acetate and subjected to the LC–MS analysis. Endosulfan lactone and Endosulfan ether were the products of degradation detected by the LCMS analysis. Plasmid curing experiments indicated that genes responsible for the degradation of Endosulfan are present on the chromosome and not on the plasmid, as growth of *Ps. aeruginosa* was observed on modified non-sulfur medium with Endosulfan after the plasmid was cured with ethidium bromide. The results of PCR indicated that there is no amplified product of ~1350 bp expected for *esd* gene, in *Ps. aeruginosa*, although there were some non-specific bands. Enzymatic degradation studies indicated that the enzymes involved in the degradation of Endosulfan are intracellular. With this investigation, it was indicated that immobilized cells of *Ps.* *aeruginosa* have the potential to be used in the bioremediation of water contaminated with Endosulfan.

## Introduction

Endosulfan is a cyclodiene organo-chlorine. It is a mixture of the two isomers, α and β-Endosulfan. Both of these isomers are toxic, with the α-isomer being more toxic than β-isomer (JenNi et al. 2005). It is used extensively throughout the world as a contact and stomach insecticide and as an acaricide on field crops, such as cotton, paddy, sorghum, oilseeds, coffee, vegetables, and fruit crops (Lee et al. 1995; Kullman and Matsumura 1996). It is used to control chewing and sucking insects, such as Colorado beetle, flea beetle, cabbage worm aphids and leaf hopper (Goebel 1982; Hoechst 1990). Endosulfan has been implicated in mammalian gonadal toxicity (Sinha et al. 1997), genotoxicity (Chaudhuri et al. 1999), and neurotoxicity (Paul and Balasubramaniam 1997).

Biodegradation of pesticides can be carried out using free as well as immobilized cells. Research has suggested that this higher productivity results from cellular or genetic modifications induced by immobilization. Evidences indicate that the immobilized cells are much more tolerant to perturbations in the reaction environment, are less susceptible to toxic substances make immobilized cell systems, and are particularly attractive for the treatment of toxic substances, such as pesticides (Manohar et al. 2001; Kim et al. 2002; Jianlong et al. 2002). Various materials have been selected as carriers for the immobilization of microorganisms to treat wastewater, including network polymers, alginate, polyacylamide hydrazide, calcium alginate, activated pumice, and activated carbon (Pai et al. 1995; Murakami et al. 2003; Pazarlioglu and Telefoncu 2005; Rahman et al. 2006; Karigar et al. 2006).

There are reports on the biodegradation of pesticides using immobilized cells (Ha et al. 2009; Yáñez-Ocampo et al. 2011; Abdel-Razek et al. 2013) and immobilized enzymes (Richins et al. 2000; Mansee et al. 2005). However, studies on the degradation of Endosulfan using immobilized cells are limited (Jo et al. 2010).

Aerobic bacteria degrading Endosulfan were isolated from contaminated sludge by Yu et al. (2012). LD-6 was one of the isolates, which was identified as *Stenotrophomonas* spp. Cell crude extract of strain LD-6 could metabolize Endosulfan rapidly, and degradative enzymes were intracellular distributed and constitutively expressed. Shivaramaiah and Kennedy (2006) studied the biodegradation of Endosulfan by a soil bacterium S3 which consistently degraded Endosulfan. Endosulfan degradation results indicated that the enzyme system responsible was probably a mono-oxygenase, converting Endosulfan to Endosulfan sulfate. Katayama and Matsumura (1993) showed that the cultures of *Trichoderma harzianum* were capable of producing Endosulfan diol as a principal metabolite. They suggested that a hydrolytic enzyme sulfatase is responsible for the indirect formation of Endosulfan diol by the hydrolysis of Endosulfan sulfate.

Genes involved in the degradation of Endosulfan have also been studied by many researchers (Weir et al. 2006; Verma et al. 2011; Vijaiyan and Rajam 2013). Sutherland et al. (2002a) reported the role of *esd* gene in degrading Endosulfan.

The batch degradation study using free cells and Ca-alginate immobilized cells of *Ps. aeruginosa* JX204836 isolated from an agricultural field was carried out by us and reported in our earlier publication, where immobilized cells showed a better degradation potential at higher pesticide concentrations compared to free cells (Vijayalakshmi and Usha 2012). The present study is taken up with the objective of studying the continuous and repeated batch degradation of Endosulfan using immobilized cells of *Ps. aeruginosa*, isolated from a soil amended with the pesticide. The research also involves the study of genes and the enzymes involved in the degradation of Endosulfan, so that the enzymes could also be immobilized and utilized for the process of bioremediation of waters contaminated with endosulfan.

## Materials and methods

### Pesticide and other chemicals

Commercial-grade insecticide Endosulfan (35 % EC) was procured from a pesticide selling shop in Bangalore. Other chemicals were procured from Hi-Media Pvt. Ltd. Mumbai. The Endosulfan standard was provided as a kind gift by IIHR, Bangalore. Other chemicals used in the preparation of modified non-sulfur medium (Siddique et al. 2003), K_2_HPO_4_, KH_2_PO_4_, NH_4_Cl, MgCl_2_·6H_2_O, CaCO_3_, FeCl_2_·4H_2_O, and trace element solution, the preparation of phosphate buffer, chemicals used for the immobilization and estimation of Endosulfan, and the solvent Ethyl acetate and methanol used in the extraction and dissolution of endosulfan were of analytical grade. These were procured from Sd Fine Chemicals Ltd., Mumbai, Maharashtra, India and Himedia Laboratories Pvt. Ltd., Mumbai, Maharashtra, India. For the Endosulfan estimation procedure, double distilled deionised water was used.

### Bacterial culture

In the present study, *Ps. aeruginosa*, isolated from an agricultural field with the previous history of pesticide application, identified based on nucleotide sequence and deposited in the gene bank with the accession number JX204836 was used.

### Growth of the culture

*Pseudomonas aeruginosa* was grown in modified non-sulfur medium (Siddique et al. 2003) containing 2.5 % Endosulfan under optimized conditions. After incubation, the bacterial cells were harvested by centrifugation at 10,000 rpm for 15 min. These cells after washing with 0.01 M Phosphate buffer (pH 7.0) were used for the immobilization experiments.

### Immobilization in Ca-alginate

Ca-alginate entrapment of *Ps. aeruginosa* was performed according to the method of Bettman and Rehm (1984). Sodium alginate (3 % w/v) was dissolved in distilled water and autoclaved at 121 °C for 15 min. Fresh bacterial pellet (3 % w/v) of *Ps. aeruginosa* was mixed in 100 mL sterilized sodium alginate solution. This mixture was extruded drop by drop into a cold sterile 0.2 M Calcium chloride solution using a sterile syringe. Gel beads of approximately 2 mm diameter were obtained. The beads were hardened by resuspending in a fresh 0.2 M Calcium chloride solution for 2 h with gentle agitation. Finally, these beads were washed with sterile distilled water and stored in 0.2 M Calcium chloride at 4 °C until further use.

### Repeated batch degradation of Endosulfan

Repeated batch degradation studies were performed to observe the long-term stability of Ca-alginate immobilized *Ps. aeruginosa* culture degrading Endosulfan. After each cycle of incubation for 24 h at 150 rpm shaking speed and at 37 °C, the spent medium was decanted, and beads were washed with sterile distilled water and transferred into a fresh sterile minimal mineral salt medium (Manohar and Karegoudar 1998) containing 2 % Endosulfan.

The remaining amount of Endosulfan in the media after incubation was estimated by spectrophotometric analysis, as described by Venugopal and Sumalatha (2011). At intervals of 5 days/cycles, the stability of beads was monitored, and cell leakage was recorded as Cfu/mL values by plating 1 mL of spent medium onto nutrient agar medium.

### Design of bioreactor for continuous treatment

**Fig. 1:**
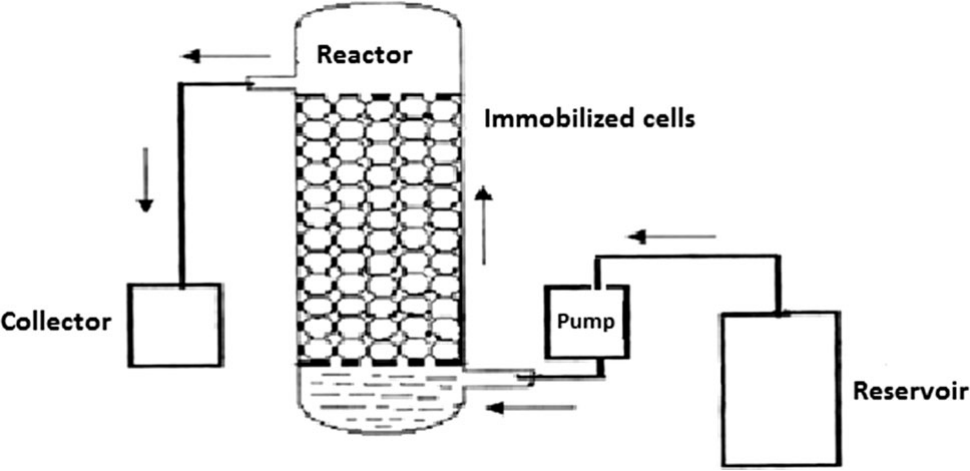
Schematic diagram of the bioreactor

**Fig. 2:**
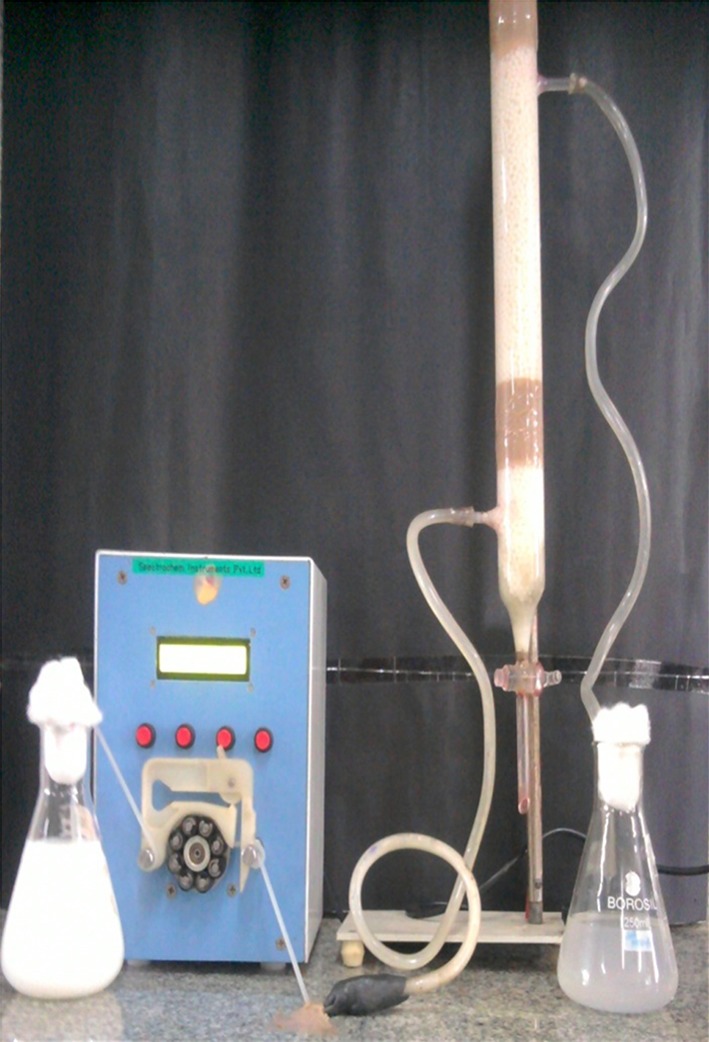
Reactor packed with Ca-alginate immobilized *Ps. aeruginosa*

A schematic representation of the cylindrical glass column used as the bioreactor for continuous degradation of Endosulfan is shown in Fig. 1. The column (4 × 50 cm volume 650 mL), as shown in Fig. 2, with inlet and outlet facilities was used. The bottom of the column was packed with glass wool (4 cm diameter) followed by a porous glass frit. Then, the reactor was packed with the Ca-alginate immobilized culture of *Ps. aeruginosa* for the degradation of the pesticide to a height of 30 cm. The reactor was attached to a reservoir containing minimal mineral salts medium (Manohar and Karegoudar 1998) with Endosulfan. The medium after pesticide degradation was continuously removed from the side arm situated just above the packed bed.

The detention time (dt) of degradation was calculated by the following formula:

1 $${\text{Detention time: void volume/flow rate }}\left( {\text{mL/h}} \right)$$

2 $${\text{Degradation rate}}\left( R \right) = \left( {C_{\text{i}} { - }C_{\text{e}} } \right) \, \times {\text{ D}}$$

where *C*_i_ = concentration of the pesticide in the influent

*C*_e_ = Concentration of the pesticide in the effluent

3 $$D={\text{Dilution rate}} = {\text{flow rate }}\left( {\text{mL/h}} \right) / {\text{ void volume of the reactor }}\left( {\text{mL}} \right)$$

where flow rate is the quantity of the sample passing through the column, expressed as mL/h.

### Continuous degradation of Endosulfan

The continuous treatment of Endosulfan was carried out in a continuous flow reactor. The reactor was filled with Ca-alginate immobilized *Ps. aeruginosa* for the degradation of Endosulfan. Degradation process was carried out by continuous supply of sterile minimal mineral salts medium containing Endosulfan with the help of peristaltic pump (Miclins PP10-4C, India). Flow rates ranged from 20 mL/h to 100 mL/h with varying concentrations of Endosulfan (2–10 %).

### Estimation of Endosulfan

Estimation of Endosulfan was carried out according to Venugopal and Sumalatha (2011). Acid reagent was prepared by dissolving 304 g of p-Toluene sulfonic acid in 1 L of isopropanol plus 200 mL of water (Raju and Gupta 1991). Aliquotes of one mL of extracts were dried, and to the aliquote, 5 mL of acid reagent was added. To this, 1 mL of 2 % alcoholic potassium hydroxide and 10 mL of 0.1 N of hydrogen peroxide were then added. The sulfur dioxide liberated was oxidized to sulfate and allowed to react with 0.1 mL of 0.1 % solution of diphenyl amine to give a light violet colour. The solution was kept aside for 5 min, and the absorbance was measured at 605 nm against reagent blank. The absorbance corresponding to the bleached colour which in turn corresponds to the analyte Endosulfan concentration was obtained by subtracting the absorbance of the blank solution from that of test solution.

### Estimation of percent degradation

Percent degradation was calculated using the given formula:

4 $${\text{Percent degradation }} = \, \left[ {\left( {C_{0} - Ct} \right)/C_{0} } \right] \, \times 100$$

where *C*_0_ = initial concentration and *Ct* = concentration at time ‘*t*’.

Degradation efficiency is defined as the ability of *Ps. aeruginosa* to degrade the pesticide and is calculated based on percent degradation as shown in Eq. (4).

### Identification of degradation products

After degradation of Endosulfan using Ca-alginate immobilized *Ps. aeruginosa*, the products were extracted from a large amount of spent medium using two volumes of ethyl acetate, dried, and finally mixed with methanol and sent to Indian Institute of Science, Bangalore for the LC–MS analysis.

### LC–MS analysis conditions

HPLC analyses were performed using Thermo Finnigan Survey. The column used was a BDS HYPERSIL C18 (Reverse Phase) with length 250 mm, I.D. 4.6 mm, and particle size 5 µm. Detection was done with UV at 254 and 280 nm. The detector used was HPLC PDA/UV detector; with ambient temperature and injection volume of 10 µL. An isocratic eluent with Acetonitrile:Water in the ratio 70:30 was used. The flow rate was 0.2 mL/min with a run time of 60.00 min. HPLC grade acetonitrile from the company Merck was used. The water used as a part of the isocratic eluent was milli-Q water.

Mass spectroscopy (MS) was performed using Thermo LCQ Deca XP MAX. The software used was Xcalibur. Conditions used for MS were—probe/source voltage of 4.5 kV; mode of ionization +ve mode; mass range 50–500 *m*/*z*; sheath gas flow (arbitrary units): 40.00; auxiliary/sweep gas flow (arbitrary units): 20.00; source type: ESI (Electro Spray Ionization); sample trey temperature: 5 °C; column oven temp: 40 °C; capillary temperature: 275 °C; capillary voltage: 16.00 V; nebulisation gas flow: helium at 1 mL/min approximately. The helium in the mass analyzer cavity was maintained at 0.1 Pa (10^−3^).

### Plasmid isolation and plasmid curing

To know the number of plasmids present and to find out whether the genes responsible for the production of enzymes involved in the degradation of Endosulfan are present on the genomic DNA or on the extra chromosomal DNA; plasmid isolation and plasmid curing experiments were performed. The culture *Ps. aeruginosa* degrading Endosulfan was grown in Luria–Bertani (LB) broth for 24 h. Plasmid DNA was extracted using the alkaline lysis method (Sambrook and Russell 2001) from the cell pellet of the culture. The extracted plasmid DNA band was observed by agarose gel electrophoresis.

*Ps. aeruginosa* degrading Endosulfan was inoculated into 100 mL nutrient broth medium and incubated for 24 h under shaking speed of 150 rpm. After 24 h of incubation, 1 mL of broth was inoculated into fresh nutrient broth with 300 µg/mL of Ethidium bromide. Plasmid DNA was extracted (Sambrook and Russell 2001) and subjected to 1 % agarose gel electrophoresis. The procedure was repeated up to 6 days. Plasmid cured culture was checked for its efficiency in degrading Endosulfan by plating 1 mL of culture on modified non-sulfur medium (Siddique et al. 2003).

### Genomic DNA isolation and PCR analysis

*Ps. aeruginosa* culture degrading Endosulfan was subjected to the PCR analysis, which was outsourced from Bhat Bio-tech India Private Limited, Bangalore. The genomic DNA was isolated from *Ps. aeruginosa* using genomic DNA extraction Kit. The pellet from 1.5 mL of overnight culture was resuspended in 500 µL of lysis buffer and incubated at 37 °C for 1 h to lyse the cells. Genomic DNA was then extracted by Phenol/Chloroform. DNA from the aqueous phase was precipitated with isopropanol, washed with 70 % Ethanol, and air dried. The DNA pellet was dissolved in 50 µL of nuclease free water. 1 µL of the genomic DNA was used to analyze on 0.5 % Agarose gel electrophoresis.

Amplification of the *esd* genes was performed using the following primer pairs Sutherland et al. 2002a)

*esd* Forward primer: 5′-CCATATGACCCGACAGCTACACCTC-3′

*esd* Reverse Primer: 5′-CAGATCTATTACGCGACCGCGTGCGCCA-3′

The amplification was carried out in a Master cycler^®^ Thermocycler (Eppendorf, Germany) using the following program. For the amplification of *esd* gene, the initial denaturation of 95 °C for 2 min followed by 35 cycles of denaturation at 94 °C for 30 s, annealing at 63 °C for 45 s and extension at 72 °C for 1 min was used. The final extension was carried out at 72 °C for 10 min. 10 µL of PCR product was analyzed on 0.8 % Agarose gel electrophoresis. The kit that was used for the analysis was from Bhat Bio-tech India Private Limited, Bangalore.

### Enzymatic degradation of Endosulfan

Experiment was conducted to find out whether the enzymes involved in the degradation of Endosulfan by *Ps. aeruginosa* were extracellular, membrane bound, or intracellular.

*Pseudomonas aeruginosa* was grown in 100 mL modified non-sulfur medium (Siddique et al. 2003) under optimized conditions. The cells of the culture from the medium were harvested during mid logarithmic phase by centrifugation at 10,000 rpm for 20 min at 4 °C. Supernatant of the culture was stored separately. The cell pellet of *Ps. aeruginosa* was washed with 1 mL of 0.05 M phosphate buffer (pH 6.8) (Yu et al. 2012). Buffer wash of the culture was collected separately. The cell pellet obtained was resuspended in 1 mL of the buffer. The cells were lysed using 1 mL of freshly prepared lysozyme solution (10 mg/mL) in 10 mM Tris–HCl, pH 8.0 (Sambrook et al. 1989). The supernatant, buffer wash, and cell lysate were refrigerated until further use. Enzymatic degradation of Endosulfan was studied with all the three fractions, i.e., supernatant, buffer wash, and cell lysate. The protein content of each fraction was determined by the Bradford’s method (1976).

Enzymatic degradation of Endosulfan was carried out according to Yu et al. (2012). The reaction mixture was taken in triplicates with 1 mL of 0.05 M phosphate buffer (pH 6.8) containing 200 µL of supernatant and 50 mg/L of Endosulfan. The reaction mixture was incubated at 30 °C for 45 min. After the incubation, the residual Endosulfan was quantified spectrophotometrically at 605 nm according to Venugopal and Sumalatha (2011). The amount of Endosulfan degraded was calculated. Similar experiments were carried out with 200 µL of the buffer wash and cell lysate separately, and the degradation of Endosulfan was recorded in both the cases. The cell lysate was subjected to ammonium sulfate precipitation and dialysis, as it showed better results compared to the other fractions.

Ammonium sulfate was added to the crude cell lysate of *Ps. aeruginosa* to give 100 % saturation. The solution was stirred at 4 °C for 30 min and centrifuged at 15,000×*g* for 20 min; the precipitate was redissolved in 0.05 M phosphate buffer of pH 6.8.

Enzymatic degradation of Endosulfan was performed using the ammonium sulfate precipitated fraction. The ammonium sulfate precipitated sample of *Ps. aeruginosa* was dialysed overnight against 100 vol of 0.05 M phosphate buffer (pH 6.8) at 4 °C. The dialysed sample was used for enzymatic degradation of Endosulfan. The protein content of ammonium sulfate precipitated and dialysed buffer wash fraction was determined by the Bradford’s method (1976).

## Results

### Repeated batch degradation

**Fig. 3:**
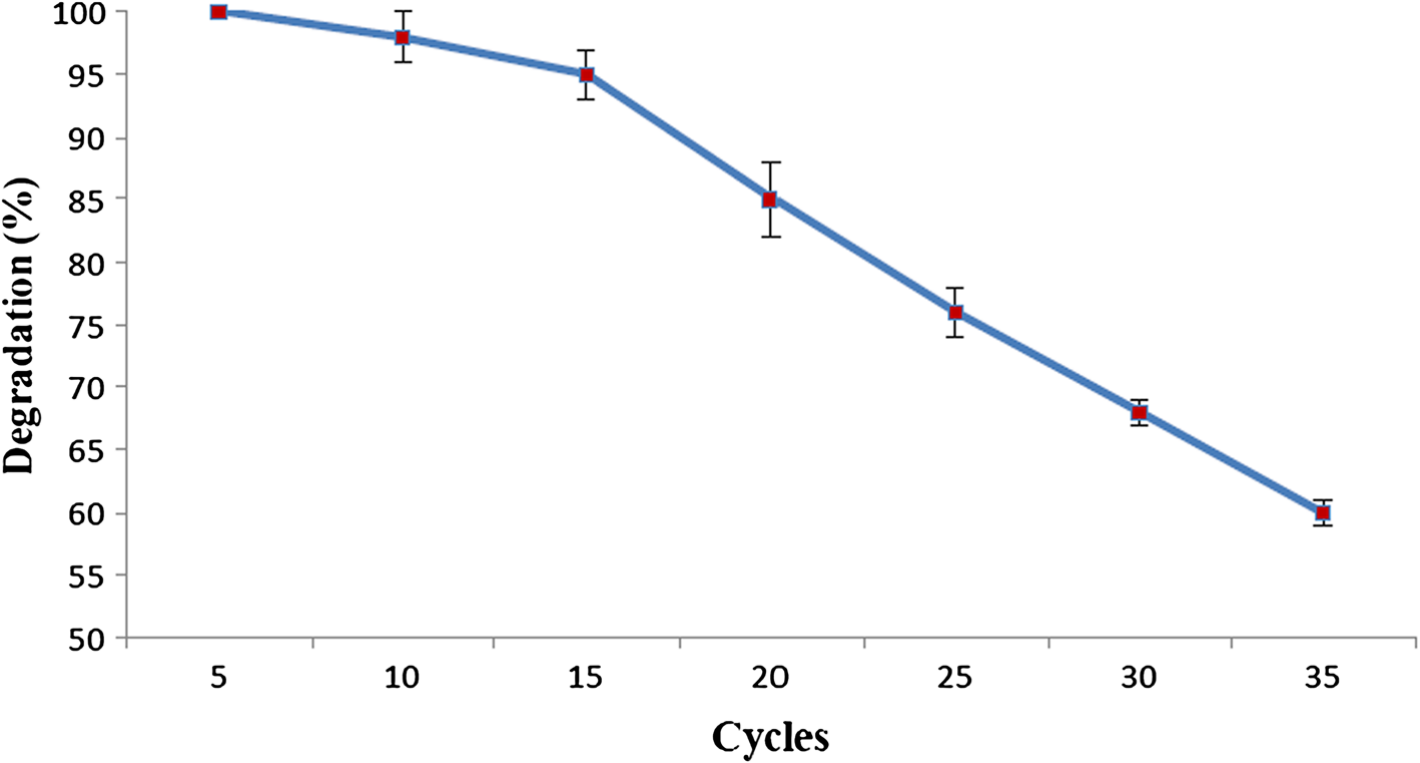
Repeated batch degradation of Endosulfan by Ca-alginate immobilized *Ps.* *aeruginosa. Error bars* represent the standard deviation of three replicates

From Fig. 3, it can be observed that Ca-alginate immobilized *Ps. aeruginosa* could be reused without affecting its degradation efficiency of 100 % up to 5 cycles. By the end of the 10th cycle, the degradation efficiency decreased only by 2 %. Furthermore, by the end of the 15th cycle, the degradation efficiency decreased to 95 %, and by the 20th cycle, it came down to 85 %. However, by the end of the 25th cycle and the 30th cycle, the degradation efficiency decreased further to 76 and 68 %, respectively. By the end of the 35th cycle, 60 % degradation efficiency was retained. The mechanical stability of the beads decreased gradually, as the cell leakage increased with cycles. A drastic reduction in the stability of the beads was noted by the 35th cycle. After 5 cycles, a cell leakage of 56 Cfu/mL was reported which increased with the number of cycles. A cell leakage of 642 × 10^4^ Cfu/mL was recorded by the end of the 35th cycle. The results of cell leakage over time are indicated in Table 1.

**Table 1:** Cell Leakage monitored every 5 cycles in repeated batch degradation

Cycle	Cell leakage (Cfu/mL)
5	56
10	379
15	775
20	295 × 10^2^
25	518 × 10^2^
30	892 × 10^3^
35	642 × 10^4^

### Continuous degradation of Endosulfan

**Fig. 4:**
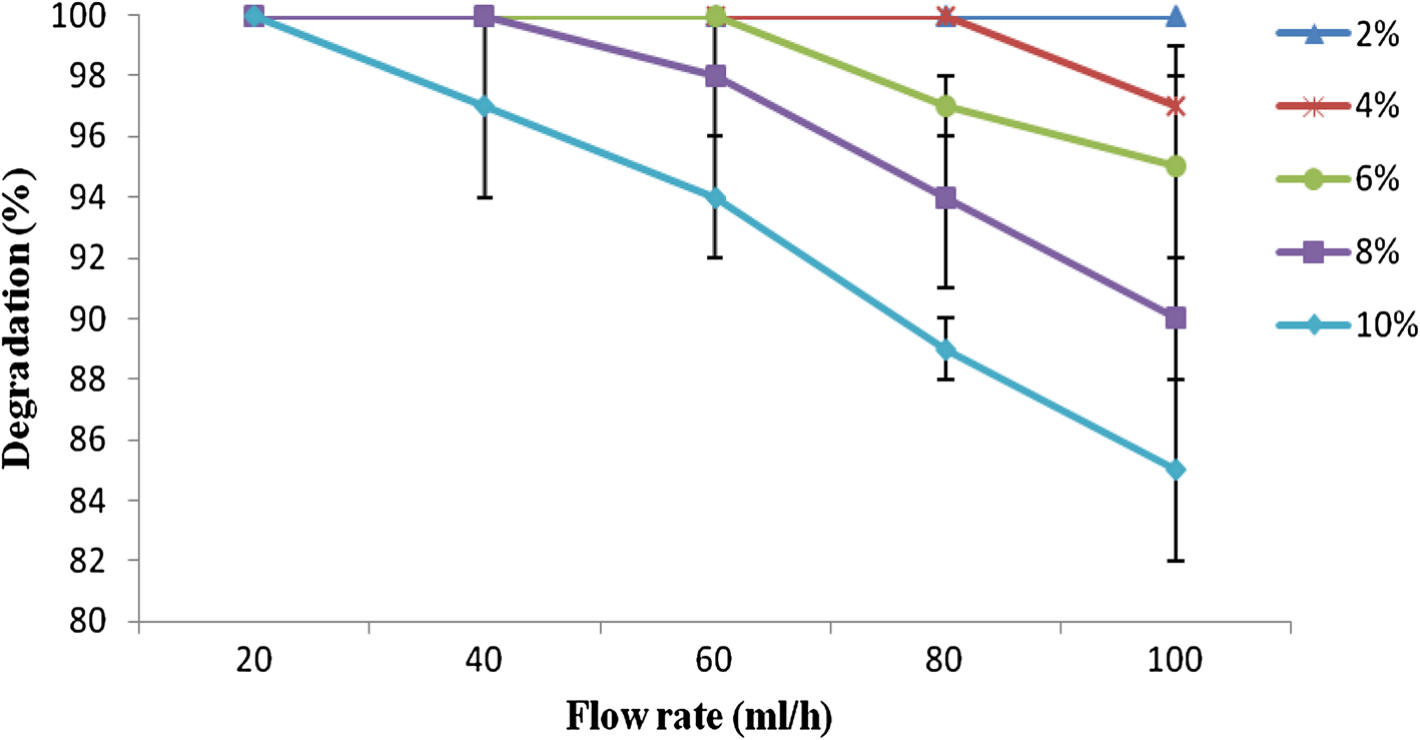
Continuous degradation of Endosulfan by immobilized *Ps. aeruginosa. Error bars* represent the standard deviation of three replicates

As shown in Fig. 4, 100 % degradation was obtained with 2 % concentration of Endosulfan up to 100 mL/h flow rate (dt 36 min) and with 4 % concentration of Endosulfan up to 80 mL/h flow rate (dt 108 min). At 20 mL/h flow rate (dt 324 min), 100 % degradation was recorded even up to 10 % concentration of pesticide. At 40 mL/h flow (dt 252 min) rate, *Ps. aeruginosa* was able to show 100 % degradation of Endosulfan up to 8 % concentration. With 10 % concentration of pesticide at 100 mL/h flow rate (dt 36 min), 85 % degradation of Endosulfan was recorded.

### LC–MS analysis

The retention time for the Endosulfan standard was found to be 23.66. Compared to the chromatogram of Endosulfan control (Undegraded), the peak at retention time 23.62 reduced considerably in the chromatogram of Endosulfan test sample (degraded) which indicates that only traces of Endosulfan are left in the test sample.

**Fig. 5:**
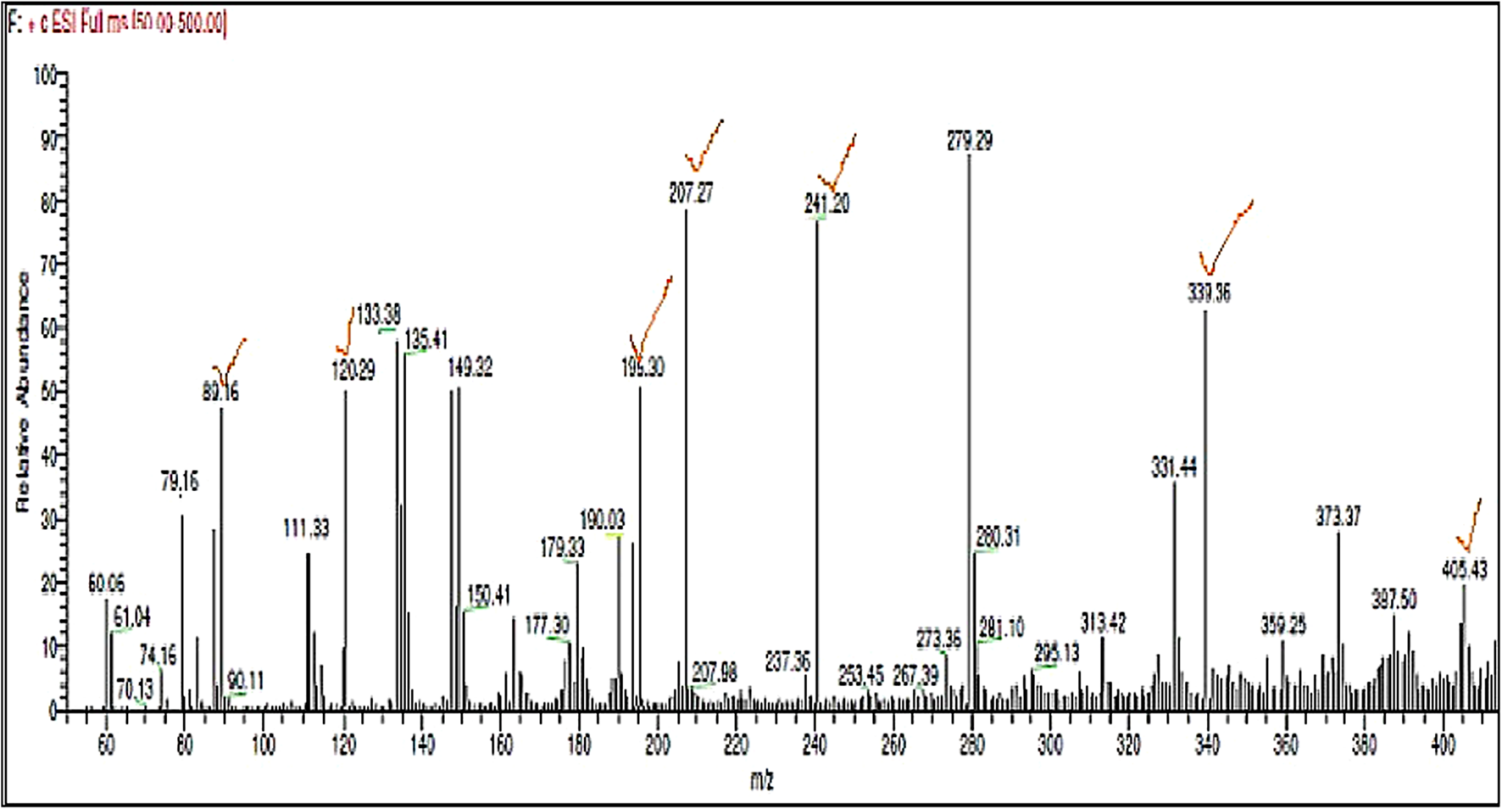
Mass spectrum of Endosulfan control, Fragment ions corresponding to Endosulfan based on NIST spectrum of Endosulfan are ticked

**Fig. 6:**
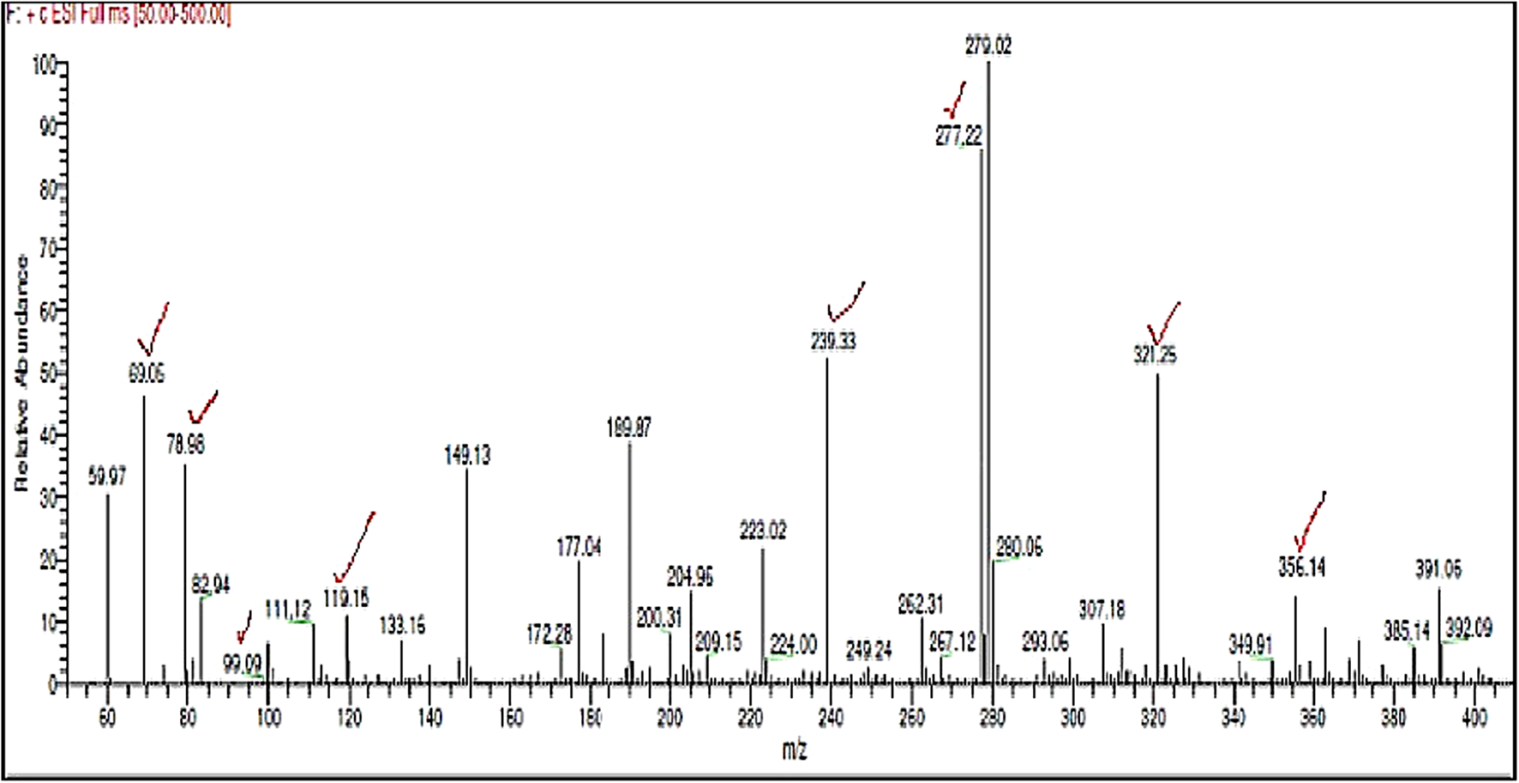
Mass spectrum of Endosulfan test, Fragment ions corresponding to Endosulfan and its degradation products, i.e., Endosulfan lactone and Endosulfan ether, based on NIST spectra are ticked

The *m*/*z* value 405.43, corresponding to the molecular weight of Endosulfan, is present in the mass spectrum of Endosulfan control (Fig. 5), but absent in the mass spectrum of Endosulfan test sample (Fig. 6).

**Fig. 7:**
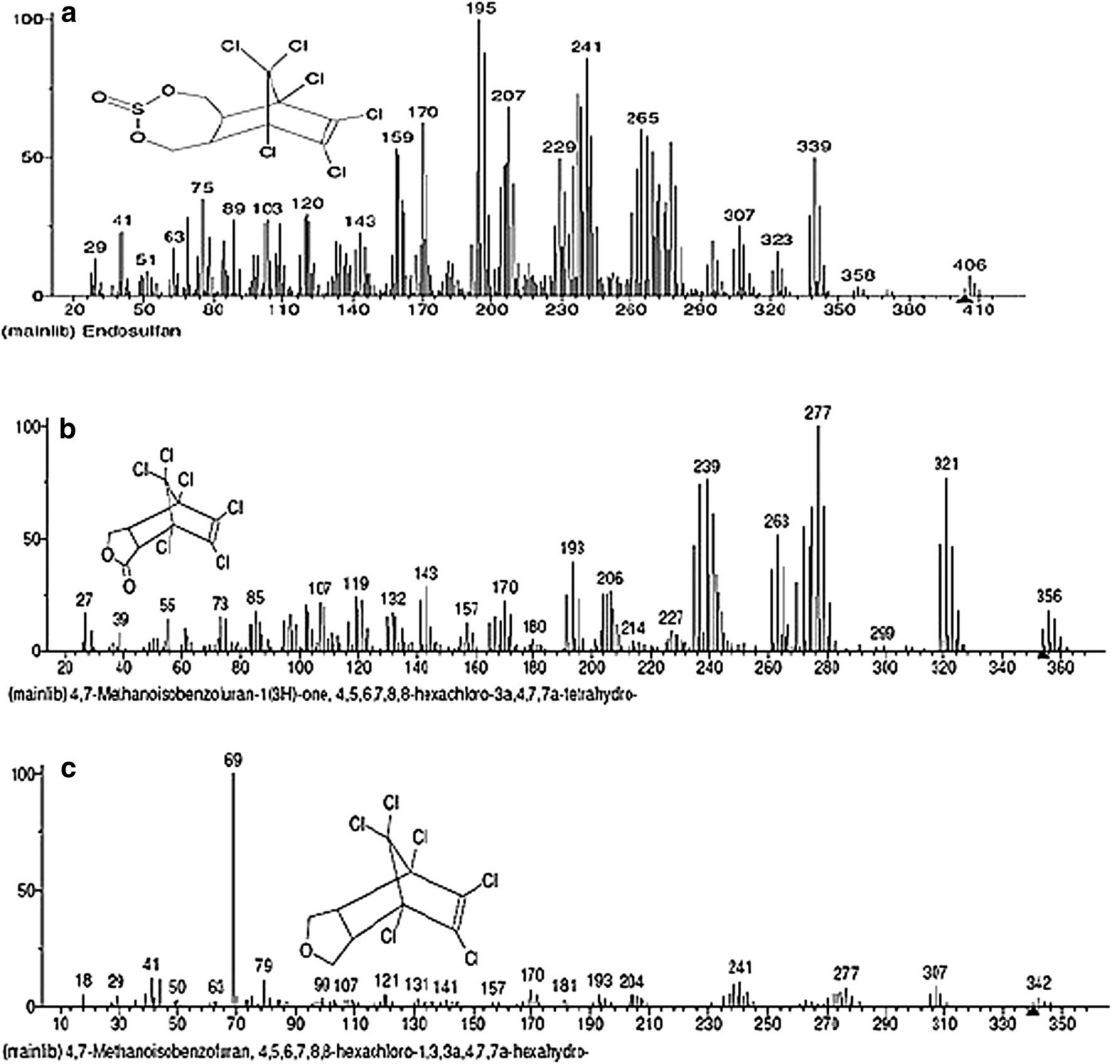
NIST standard spectra of **a** Endosulfan, **b** Endosulfan Lactone, **c** Endosulfan ether (Contributor: Catalogue of mass spectra of pesticides, April, 1975; J. Freudenthal & L. G. Gramberg, Nat’l Inst. of Public Health, The Netherlands)

The *m*/*z* values corresponding to Endosulfan in the mass spectrum of Endosulfan control sample and mass spectrum of test sample according to the NIST standard (Fig. 7a) are listed in Table 2. In the mass spectrum of the Endosulfan test sample, *m*/*z* peaks corresponding to Endosulfan lactone and endosulfan ether according to the NIST standard (Fig. 7b, c) are seen which are listed in Table 3.

**Table 2:** *m*/*z* values of Endosulfan seen in the mass spectrum of Endosulfan control and test samples

*m*/*z* of Endosulfan in mass spectrum of Endosulfan control according to NIST standards, as shown in Fig. 7a	*m*/*z* of Endosulfan in mass spectrum of Endosulfan test according to NIST standard, as shown in Fig. 7a
**405.43**, 339.96, 280.31, 279.29, 241.20, 207.27, 195.30, 120.29, 119.22, 111.33, 89.16	307.18, 280.06, 279.02, 277.22, 189.87, 119.15, 111.12, 99.09, 82.94, 78.98

**Table 3:** *m*/*z* values of degradation products of Endosulfan in the mass spectrum of Endosulfan test sample

*m*/*z* of Endosulfan lactone according to NIST standard, as shown in Fig. 7b	*m*/*z* of Endosulfan ether according to NIST standard, as shown in Fig. 7c
356.14, 321.25, 277.22, 239.33,119.15	279.02, 277.22, 267.12, 209.15, 204.96, 172.28, 133.16, 69.06

In the mass spectrum of Endosulfan test sample (Fig. 6), *m*/*z* value of 356.14, corresponding to molecular weight of Endosulfan lactone, is seen which is absent in the mass spectrum of Endosulfan control sample (Fig. 5). The base peak of 279 is stable which is seen in both test and control samples. Along with Endosulfan lactone, additional fragments of the metabolite, Endosulfan ether, according to the NIST standard (Fig. 7c), are also present in the mass spectrum of Endosulfan test present, which are also shown in Table 3. The base peak of Endosulfan ether with *m*/*z* value of 69.06 is present in the mass spectrum of test sample (Fig. 6), but absent in the mass spectrum of control sample (Fig. 5).

### Plasmid isolation and plasmid curing

On plasmid isolation and gel electrophoresis, it was observed that *Ps. aeruginosa* has a single band of plasmid. Curing experiment on *Ps. aeruginosa* was carried out to determine the presence of Endosulfan degrading genes on plasmid or on chromosome. With the increase in incubation period, the thickness of the plasmid band on agarose gel decreased.

**Fig. 8:**
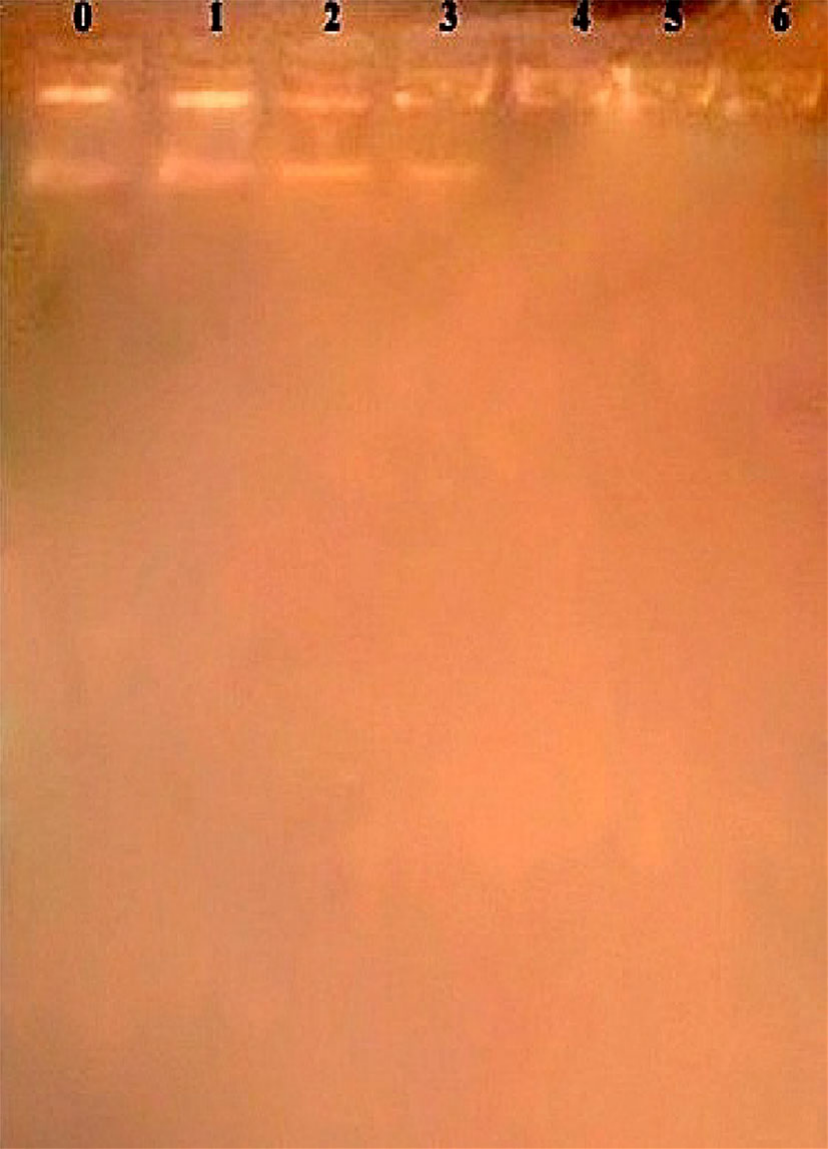
Agarose gel electrophoresis of plasmid from *Ps. aeruginosa* subjected to curing. *lane 0* indicates control sample (not treated with ethidium bromide), and *lanes 1–6* indicate ethidium bromide treated bands of plasmid extracted from days 1–6

As seen in Fig. 8, the thickness of the plasmid band reduced by day 3 and by day 4, the band disappeared indicating that the plasmid band was cured. Plasmid DNA cured cells of *Ps. aeruginosa* were able to grow on modified non-sulfur medium supplemented with Endosulfan as the sole source of carbon. This indicated that the genes responsible for the degradation Endosulfan are present on the chromosome and not on the plasmid.

### Genomic DNA isolation and PCR analysis

**Fig. 9:**
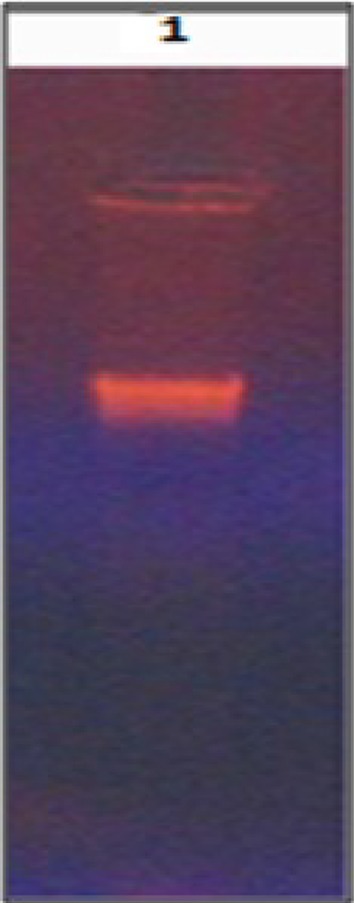
0.5 % Agarose gel electrophoresis of genomic DNA from *Ps. aeruginosa*

Genomic DNA from *Ps. aeruginosa* degrading Endosulfan was isolated, as shown in Fig. 9.

**Fig. 10:**
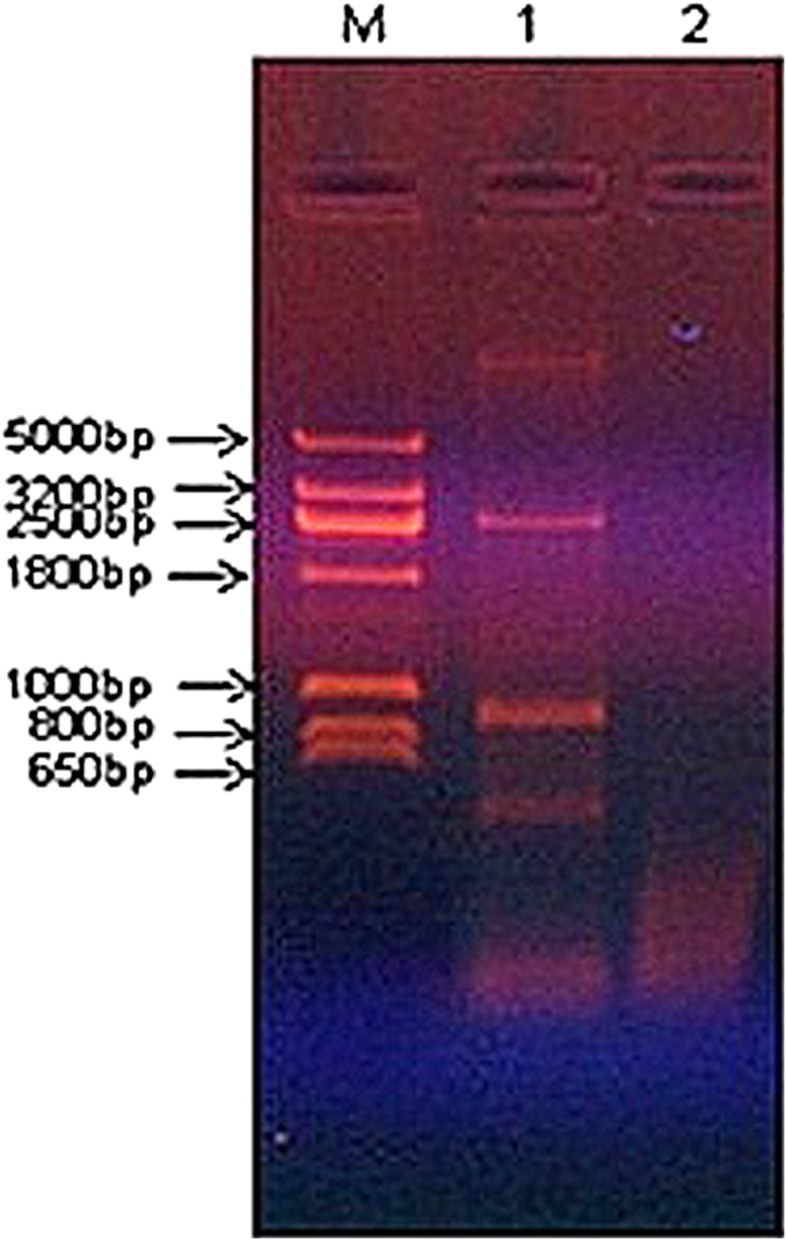
0.8 % Agarose gel electrophoresis of products of *esd* PCR. *M* DNA molecular weight marker; *1* *Ps. aeruginosa* and *2* no template control

The culture was subjected to the PCR analysis. The results of PCR indicate that there is no amplified product of ~1350 bp expected for *esd* gene, in *Ps. aeruginosa*. However, there were some non-specific bands of 5000, 3200, 2500, 1800, 1000, 800, and 650 bp. These bands could be amplified products of genes other than *esd* gene (Fig. 10).

### Enzymatic degradation of Endosulfan

The results of the enzymatic degradation of Endosulfan with the supernatant, buffer wash, and cell lysate of *Ps. aeruginosa* are presented in Table 4.

**Table 4:** Enzymatic degradation of Endosulfan with the supernatant, buffer wash, and cell lysate of *Ps. aeruginosa*

Enzyme fraction	Percent degradation of Endosulfan	Protein content (mg/mL)
Supernatant	3 ± 1	0.09 ± 0.01
Buffer wash	8.16 ± 0.76	0.343 ± 0.025
Cell lysate	15 ± 2	0.663 ± 0.03

Values are a mean of three replicates ±SD

Highest degradation of Endosulfan was recorded with crude cell lysate. Lesser degradation of Endosulfan was recorded with buffer wash and a least degradation with cell free supernatant. This indicated that the enzymes involved in the degradation of Endosulfan are intracellular.

When cell lysate of *Ps. aeruginosa* was subjected to ammonium sulfate precipitation, there was an increase in the degradation of Endosulfan. Degradation of Endosulfan increased further after dialysis of ammonium sulfate precipitated sample (Table 5).

**Table 5:** Enzymatic degradation of Endosulfan by partially purified enzyme fractions

Enzyme fraction	Percent degradation	Protein content (mg/mL)
Cell lysate of *Ps. aeruginosa*	15 ± 2	0.663 ± 0.03
After ammonium sulfate precipitation	34.33 ± 1.52	0.358 ± 0.025
After dialysis	45 ± 1	0.3 ± 0.01

Values are a mean of three replicates ±SD

## Discussion

In the current study, when repeated batch degradation study was carried out, immobilized cells of *Ps. aeruginosa* were able to show 60 % degradation of Endosulfan at the end of the 35th cycle with a cell leakage of 642 × 10^4^ Cfu/mL. In similar studies, Endosulfan degrading ability of *Klebsiella oxytoca* KE-8 immobilized by entrapment with activated carbon was investigated by Jo et al. (2010). Activated carbon with immobilized cells *K. Oxytoca* KE-8 that had been used for one degradation activity was stored for 1, 15, and 30 days at 4 °C. Endosulfan degradation activity after one month duration was 81 %, compared to 98 % before storage.

Immobilized cells have been used for the degradation of various pesticides. In the current study, a degradation of 100 % was obtained with 2 % concentration of Endosulfan up to 100 mL/h flow rate and with 4 % concentration of Endosulfan up to 80 mL/h flow rate. With 10 % concentration of pesticide at 100 mL/h flow rate, 85 % degradation of Endosulfan was recorded. Recycle packed bed column mode and continuous packed bed column mode for Endosulfan degradation were also studied by Jo et al. (2010). The effect of the hydraulic retention time (from 25 to 300 min) on the performance of bioreactor was studied by them with variation in the influent flow rate. The immobilized cells in a laboratory scale pack bed column with support beads were able to degrade Endosulfan completely in defined minimal salt medium at a maximum rate of 129.6 μg/mL per day, under optimum operation condition.

Column studies with calcium alginate entrapped cells of *Pseudomonas fluorescens* was carried out by Jesitha et al. (2015). They reported that broth mineral medium was supplemented with 350 μg/L endosulfan and was passed through the column at the rate of 0.59 mL/min and the column was operated for 18 days. At this rate, the hydraulic retention time reported was 300 min. The studies reported that the integrity of the beads was maintained.

Similar studies were also carried out by Yañez-Ocampo et al. (Yáñez-Ocampo et al. 2011) using a tezontle-packed up-flow reactor (TPUFR) with an immobilized bacterial consortium for biological treatment of methyl-parathion and tetrachlorvinphos. In the bioreactor, four flow rates (0.936, 1.41, 2.19, and 3.51 l/h) and four hydraulic residence times (0.313, 0.206, 0.133, and 0.083 h) were evaluated. With an operating time of 8 h and a flow of 0.936 l/h, they obtained 75 % efficiency in the removal of methyl-parathion and tetrachlorvinphos.

To create an *E. coli* expression construct, Sutherland et al. (2002a) amplified the *esd* gene by PCR using pYUB415-Apa3 as the template. However, in the current study, when the genome of *Ps. aeruginosa* was subjected to PCR for the amplification of *esd* gene, there was no amplified product of ~1350 bp expected to be involved in the oxidative pathway of degradation of Endosulfan in the culture. The absence of *esd* gene product could mean that *ese* gene may be present in *Ps. aeruginosa*. A gene, *ese*, encoding an enzyme capable of degrading both isomers of Endosulfan and endosulfate was isolated from *Arthrobacter* by Weir et al. (2006). The enzyme belongs to the two-component flavin-dependent mono-oxygenase family, whose members require reduced flavin for activity. An overview of aerobic endosulfan degradation by bacteria and fungi, and a summary of recent advances and prospects in this research field were given by Kataoka and Takagi (2013). Pathway summarized by the authors indicated difference in products accumulated when the degradation occurs with the involvement of *ese* and *esd* genes. According to the authors, *ese* indicated the pathway described by Weir et al. (2006) involving *ese* mono-oxygenase, whereas *esd* indicated the pathway described by Sutherland et al. (2002b) involving *esd* mono-oxygenase.

According to Seralathan et al., only the human CYP (cytochrome P450) enzymes and two bacterial genes (*esd* and *ese*) have been reported for endosulfan biotransformation (Kahli et al. 2006; Sean et al. 2008; Sutherland et al. 2002a). Thus, the genetic information on endosulfan metabolism is scanty, and it is a major setback for endosulfan biotransformation.

The other possibility in our present study could be the absence of both *esd* and *ese* gene products. In similar studies, PCR primers pairs coding for flavin mononucleotide-dependent mono-oxygenase present in a Mycobacterium spp. (*esd*) and mono-oxygenase of *Arthrobacter* spp. (*ese*) were employed by Bajaj et al. (2010) to amplify 1347-bp and 1428-bp, respectively. Genomic DNA of IITR01 as a template with the primer, pairs (*esd*1 and *esd*2) internal to the *esd* gene did not yield the expected gene product size 780 and 620, respectively; instead, a fragment of 600 bp was obtained from the genomic DNA of strain IITR01. The *ese* mono-oxygenase, which is 1428 bp, was also found to be absent, as they could obtain a non-specific PCR product of 682 bp. Later studies by them revealed that the nucleotide sequences belonged to transporter protein family.

Our current investigation indicates the fact that Endosulfan degradation by the organism is probably by the non-oxidative, alternate pathway. This observation is supported by the fact that predicted products of Endosulfan degradation in the current study by *Ps. aeruginosa* are Endosulfan ether and Endosulfan lactone, which are the products seen in the non-oxidative pathway of Endosulfan degradation. Although the first intermediate, endosulfan diol was not detected by us, since further metabolites were detected, such a pathway is proposed. The absence of the metabolites endosulfan sulfate and Endosulfan mono alcohol further confirms the presence of non-oxidative pathway. The non-oxidative pathway shows the presence of relatively non-toxic metabolites compared to the parent compound and the metabolites, such as endosulfan sulfate, seen in other pathways. Similar results were obtained by Yu et al. (2012). In their studies, Endosulfan diol and Endosulfan ether were detected as major metabolites, which indicated that the bacterium *Stenotrophomonas* spp. LD-6 might degrade Endosulfan by a non-oxidative pathway. They reported that 100 mg/L Endosulfan was completely degraded within 10 days, and Endosulfan diol and Endosulfan ether were detected as major metabolites. Kumar et al. (2007), also reported that the hydrolysis of endosulfan in some bacteria (*Pseudomonas aeruginosa* and *Burkholderia cepaeia*) yields the less toxic metabolite endosulfan diol. The diol can be converted to endosulfan ether (Hussain et al. 2007) or endosulfan hydroxyether (Lee et al. 2003) and then endosulfan lactone (Awasthi et al. 2003). Hydrolysis of endosulfan lactone yields endosulfan hydroxycarboxylate (Walse et al. 2003).

**Fig. 11:**
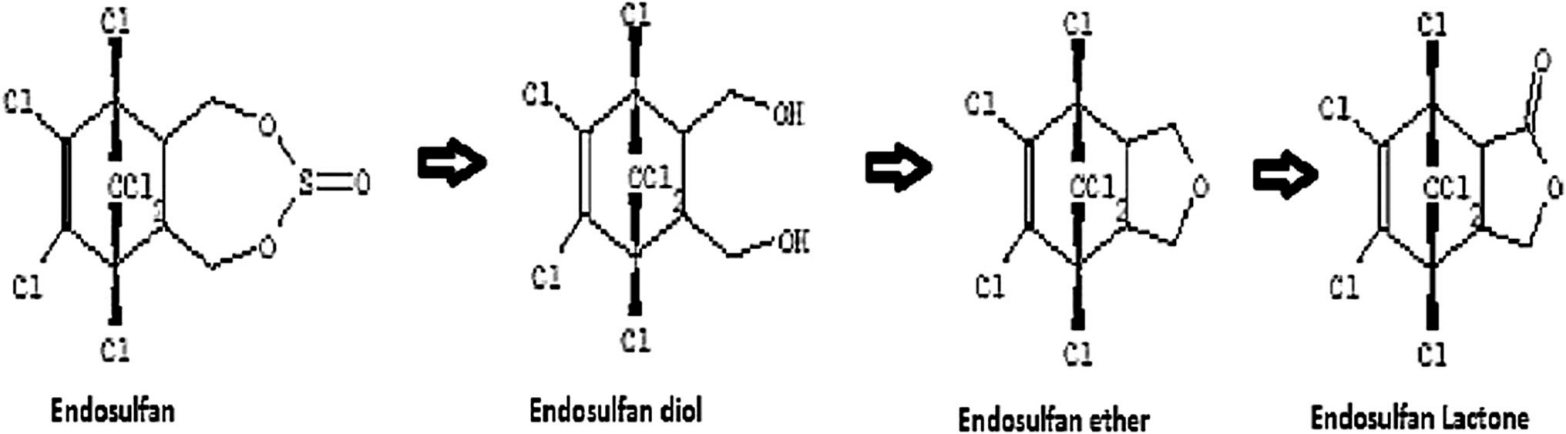
Proposed pathway of Endosulfan degradation

Based on our degradation studies, LC–MS of degradation products, and PCR analysis, the non-oxidative pathway for degradation of Endosulfan was predicted, which is shown in Fig. 11. Here, it is proposed that Endosulfan, which is a cyclodiene organo-chlorine, is converted to a less toxic intermediate, the endodiol form, and Endosulfan diol, which is further converted to Endosulfan ether and then to Endosulfan lactone. This pathway could involve the enzymes, such as hydrolases and mono-oxygenases, which need to be further studied. Similar results have been reported by Li et al. (2009) that the formation of Endosulfan diol and Endosulfan ether as the major metabolites of Endosulfan degradation by *Achromobacter xylosoxidans* CS5 and suggested a non-oxidative pathway of degradation. Bajaj et al. (2010) reported the formation of hydroxylated products ES diol, Endosulfan ether, and Endosulfan lactone by a *Pseudomonas* spp. strain IITR01.

Endosulfan degradation was better with cell lysate of *Ps. aeruginosa* compared to buffer wash and supernatant. Similar results were obtained by Yu et al. (2012), who demonstrated that the cell crude extract of strain LD-6, *Stenotrophomonas* spp. could metabolize Endosulfan rapidly and degradative enzymes were intracellularly distributed.

## Conclusion

Thus, it can be concluded that Ca-alginate immobilized cells of *Ps. aeruginosa* were efficient in degrading Endosulfan, and the Ca-alginate immobilized cells were stable for a long duration. Endosulfan was degraded by non-oxidative pathway, and Endosulfan ether and Endosulfan lactone were the predicted products of degradation. Furthermore, it was demonstrated that genes involved in the degradation of Endosulfan were present on the chromosome, and the enzymes involved in its degradation were intracellular.
